# Are sequential compression devices routinely necessary following enhanced recovery after thoracic surgery?

**DOI:** 10.1093/icvts/ivac165

**Published:** 2022-07-13

**Authors:** Sami Aftab Abdul, Caitlin Anstee, Patrick J Villeneuve, Sebatien Gilbert, Andrew J E Seely, Sudhir Sundaresan, Donna E Maziak

**Affiliations:** Department of Biology, Faculty of Science, University of Ottawa, Ottawa, ON, Canada; Division of Thoracic Surgery, The Ottawa Hospital, Ottawa, ON, Canada; Clinical Epidemiology Program, Ottawa Hospital Research Institute, Ottawa, ON, Canada; Division of Thoracic Surgery, The Ottawa Hospital, Ottawa, ON, Canada; Clinical Epidemiology Program, Ottawa Hospital Research Institute, Ottawa, ON, Canada; Division of Thoracic Surgery, The Ottawa Hospital, Ottawa, ON, Canada; Clinical Epidemiology Program, Ottawa Hospital Research Institute, Ottawa, ON, Canada; Department of Surgery, Faculty of Medicine, University of Ottawa, Ottawa, ON, Canada; Division of Thoracic Surgery, The Ottawa Hospital, Ottawa, ON, Canada; Clinical Epidemiology Program, Ottawa Hospital Research Institute, Ottawa, ON, Canada; Department of Surgery, Faculty of Medicine, University of Ottawa, Ottawa, ON, Canada; Division of Thoracic Surgery, The Ottawa Hospital, Ottawa, ON, Canada; Clinical Epidemiology Program, Ottawa Hospital Research Institute, Ottawa, ON, Canada; Department of Surgery, Faculty of Medicine, University of Ottawa, Ottawa, ON, Canada; School of Epidemiology and Public Health, Faculty of Medicine, University of Ottawa, Ottawa, ON, Canada; Division of Thoracic Surgery, The Ottawa Hospital, Ottawa, ON, Canada; Clinical Epidemiology Program, Ottawa Hospital Research Institute, Ottawa, ON, Canada; Department of Surgery, Faculty of Medicine, University of Ottawa, Ottawa, ON, Canada; Division of Thoracic Surgery, The Ottawa Hospital, Ottawa, ON, Canada; Clinical Epidemiology Program, Ottawa Hospital Research Institute, Ottawa, ON, Canada; Department of Surgery, Faculty of Medicine, University of Ottawa, Ottawa, ON, Canada; School of Epidemiology and Public Health, Faculty of Medicine, University of Ottawa, Ottawa, ON, Canada

**Keywords:** Thoracic surgery, Enhanced recovery, Sequential compression devices, Venous thromboembolism

## Abstract

**OBJECTIVES:**

The prominence of “enhanced recovery after surgery” (ERAS) protocols being adopted in thoracic surgery requires a re-evaluation of mechanical venous thromboembolism (VTE) prophylaxis guidelines. The goal of this study was to assess the role of sequential compression devices (SCD) in the prevention of VTEs such as deep vein thrombosis and pulmonary embolism (PE) in thoracic surgical patients.

**METHODS:**

We identified 200 patients who underwent elective oncological thoracic surgery between December 2018 and December 2020 in 2 cohorts—1 with SCDs and 1 without (i.e. non-SCD). All patients followed a standardized enhanced recovery after surgery (ERAS) protocol. The quality of care provided by SCDs was evaluated by the incidence and severity of postoperative and follow-up VTEs. Cohorts were compared by the Caprini score (CS) and the Charlson Comorbidity Index (CCI) with a two one-sided *t*-test analysis. Secondary outcomes include perioperative characteristics and follow-up data.

**RESULTS:**

Only 2 patients within the SCD group developed a PE with average CS and CCI metrics, both after hospital discharge and treated with anticoagulants, raising concern over the prophylactic nature of SCDs. The CS (6.9 ± 1.3 and 6.9 ± 1.5; *P *=* *0.96) and the CCI (3.8 ± 2.0 and 4.1 ± 2.6; *P *=* *0.33) for non-SCD and SCD, respectively, did not differ. The two one-sided t-test analysis for CS (*P *<* *0.001) and CCI (*P *<* *0.001) demonstrated equivalence.

**CONCLUSIONS:**

Although larger studies are required to confirm these results, routine SCD use may not be required when implementing ERAS protocols because clinically significant VTE rates were minimal.

**Figure:**
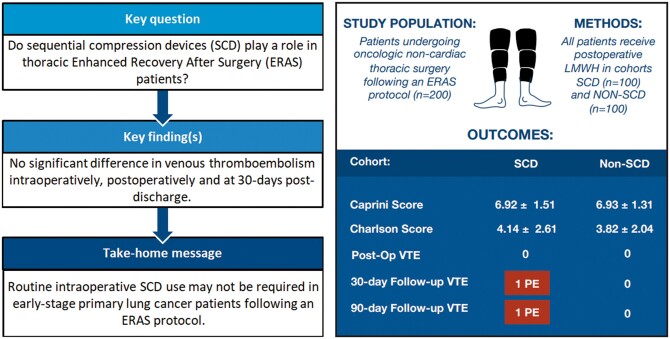

## INTRODUCTION

The implementation of Enhanced Recovery After Surgery (ERAS) protocols in all surgical specialties is rising in prominence, involving an interdisciplinary team focused on integrating perioperative evidence-based medicine into clinical practice. The literature surrounding the recommendation of mechanical venous thromboembolism (VTE) prophylaxis in thoracic ERAS protocols remains limited. The incidence and causes of VTE such as deep vein thrombosis (DVT) and pulmonary embolism (PE) are characterized as morbidity of the surgical stress response [1]. Changes in neural, endocrine and metabolic systems can induce activation of the compensatory stress response following surgery, promoting coagulation-fibrinolytic dysfunction in which the development of symptomatic or asymptomatic thromboembolic risk increases [1]. The current mainstay in addressing embolic risk involves implementing recommendations set out by the American College of Chest Physicians–9th edition VTE prophylaxis guideline and the British National Institutes of Health Care and Excellence (NICE)–VTE NG89 guideline [2, 3]. However, they report weak consensus and evidence on the incremental benefit of the routine usage of sequential compression devices (SCDs) for VTE risk in the context of the ERAS thoracic surgery pathway of care [4, 5].

The American College of Chest Physicians and the National Institute of Health and Care Excellence VTE prophylaxis guidelines support the administration of low-dose unfractionated heparin or low-molecular-weight heparin (LMWH), in addition to mechanical prophylaxis such as applied compression devices in moderate-high risk patients with VTE [4]. There is moderate to low-quality evidence to support these guidelines in thoracic surgery, and data are often extrapolated from similar surgical fields with a strong precautionary recommendation favouring anticoagulants over mechanical compression. Enhanced Recovery After Surgery (ERAS) or Enhanced Recovery After Thoracic Surgery protocols are designed to reduce the surgical stress response [4]. Similar initiatives set out earlier in the field of colorectal surgery such as “early pathway” or “fast-track rehabilitation” captured the advantages of this pathway protocol to emphasize quality rather than speed of recovery [6–10]. Guidelines for lung cancer surgery ERAS have been assembled by the European Society of Thoracic Surgeons, from which they developed 45 ERAS recommendations to mediate concomitant morbidities from initial presentation to postoperative discharge and follow-up [4]. The key recommendations of ERAS include early ambulation, smoking cessation, nutritional screening, carbohydrate loading, VTE prophylaxis, minimally invasive approach, use of antibiotics, dynamic pain relief, early chest drain removal and avoidance of urinary catheters, to name a few [4, 6].

Despite the prominence of ERAS, there is sparse evidence assessing quality provided by SCDs in strategic pathways of care. Knight and Dawson’s hallmark study elucidated the mechanism and efficacy of SCDs [9]. However, in the era of pharmacological VTE prophylaxis and ERAS pathways, the role of SCDs is becoming narrower. Several thoracic surgical and thrombosis societies further echo the limited literature from thoracic surgery in antithrombotic guideline formation [5]. These compression devices cost CAN$53 per pair at our institution. They have become an incremental expense and burdensome recommendation in this patient population following the precautionary application based on preoperative VTE risk assessment models such as the Caprini score. With the introduction of perioperative multimodal interventions and their associated superior outcomes, such as minimal postoperative morbidities with a reduction in length of stay (LOS) to 2–3 days [10, 11], there is evermore a reason to further optimize these pathways. This study assesses routine intraoperative SCD use in the prevention of VTEs in thoracic surgical oncology patients following an ERAS protocol.

## PATIENTS AND METHODS

### Ethical statement

The Ottawa Health Science Network–Research Ethics Board and the Ottawa Hospital Research Institute approved the collection of thoracic patient chart and record review of data through waived consent (approval number: 20190671-01H).

### Development and classification of patient cohorts and data management

Patients undergoing elective oncological non-cardiac thoracic surgery (December 2018 to December 2020) were identified retrospectively through the Thoracic Surgery Quality Monitoring, Information Management, and Clinical Documentation software system [12]. Patients under the age of 18 years and emergency cases were excluded. The decision to administer SCDs is made at the surgeon’s discretion and is applied intraoperatively on both legs based on factors such as prior venous disease or clotting disorder, obesity (body mass index > 40), prolonged duration of surgery and the complexity of the operation (i.e. oesophagectomy). Patients meeting these criteria and classified as SCD or non-SCD (NSCD) were recruited on a consecutive sampling non-matched basis until 100 patients in each cohort were reached. All patients followed an ERAS pathway of care and received postoperative LMWH as per the clinical pathway, at 08:00 a.m. on postoperative day (POD) 1. Patients received enoxaparin 40 mg subcutaneously once every 24 h. If creatine clearance was less than 30 ml/min, enoxaparin at 30 mg subcutaneously once every 24 h or heparin at 5000 units subcutaneously once every 8 h was administered.

### Venous thromboembolism risk and comorbidity assessment models

The Caprini score (CS) computes and stratifies postoperative embolic complications based on predisposing risk factors [13] and is validated across multiple surgical subspecialties [14–18]. The Charlson Comorbidity Index (CCI) is a validated tool for assessing the 10-year mortality risk based on patient comorbidities, excluding clinical and surgical risk factors [19]. CCI identifies comorbid risk factors not accounted for by the Caprini model while describing cohort diversity. Higher CCI scores have been correlated with an increased risk of VTE [2].

### Statistical analysis

Continuous variables were analysed using a Student *t-*test (two-tailed) and categorical values, using Fischer’s exact test (two-tailed) according to the presence and absence of SCDs. A two one-sided I-test (TOST) equivalence analysis evaluated non-significant primary measures (i.e. CS, CCI) [20]. The study was underpowered to detect asymptomatic and symptomatic VTEs; however, the sample size was sufficient for the equivalence study design. A Cohen’s d standardized effect size of 0.49 to 0.49 was set a priori to determine the raw mean difference equivalence bounds in both the CS and CCI with a power of 94%. The TOST analysis performs *t*-tests for the upper (Δ_U_) and lower (Δ_L_) margins against the raw mean difference, whereas a 90% or 95% confidence interval (CI) between these margins indicates cohort equivalence. There was no prespecified plan to adjust for multiple comparisons. Statistical significance was determined at a P-value less than 0.05 for all analyses using SPSS (version 27, IBM, Armonk, NY, USA) or R Studio (Version 1.3.1091, 2020 PBC, Boston, MA) statistical software.

## RESULTS

### Patient demographic characteristics and medical history

Table 1 showcases a comparable distribution of risk factors with no major statistically significant differences aside from obstructive sleep apnea (*P *=* *0.005) and preoperative radiation (*P *=* *0.011). Demographic characteristics such as sex, age and body mass index are held comparable across cohorts. There is a difference in former (*P *=* *0.001) and current smoking status (*P *=* *0.018); however, pack year exposure remains consistent (*P *=* *0.18). In each cohort, 42–44% of patients report a history of cancer. On average all patients had satisfactory pulmonary compliance and creatinine levels.

**Table 1:** Patient demographic characteristics and medical history

Characteristic^a^	Non-SCD (n = 100)	SCD (n = 100)	*P* Value
Sex			0.88
Male	36	38	
Female	64	62	
Age, years	67.48 ± 9.73	66.87 ± 9.27	0.65
Age categories			
<65 years	26	38	0.095
65–74 years	54	38	0.033
75–84 years	19	23	0.60
>84 years	1	1	1.0
BMI, kg/m^2^	27.37 ± 6.04	27.78 ± 11.71	0.75
Medical history			
Cerebrovascular accident	0	2	0.49
Myocardial infarction	2	4	0.68
Hypertension	51	41	0.20
High blood pressure	5	4	1.0
COPD	27	22	0.51
Pneumonia <3 months prior	0	0	…
Asthma	13	11	0.82
Obstructive sleep apnoea	13	2	0.005
Cerebrovascular disease	1	3	0.62
Diabetes	14	20	0.34
Insulin dependent	1	5	0.21
Melanoma	0	3	0.24
Sickle cell	0	0	…
Von Willebrand disease	0	0	…
Factor deficiencies	0	1	1.0
Preoperative chemotherapy	6	12	0.21
Preoperative radiation	5	17	0.011
Family history of VTE	3	1	0.62
Contraceptives	0	0	…
Anticoagulants	2	4	0.68
Coumadin	1	0	1.0
Varicose veins	0	0	…
Smoking status			
Never	20	30	0.14
Former	71	48	0.001
Current	9	22	0.018
Pack years	22.33 ± 27.78	17.79 ± 19.88	0.18
Previous cancer	42	44	0.88
FEV1^b^	85.67 ± 18.41	88.06 ± 24.84	0.45
DCLO^b^	73 ± 17.18	73.31 ± 18.31	0.90
Creatinine, mmol/l^b^	74.21 ± 30.13	86.92 ± 100.84	0.24

a Categorical and continuous data are presented as n or mean ± SD, respectively.

b 4 patients in the NSCD and 8 patients in the SCD cohorts did not receive pulmonary function tests or creatinine laboratory testing and were omitted from this analysis.

BMI: body mass index; COPD: chronic obstructive pulmonary disease; DCLO: diffusing capacity of the lung for carbon monoxide; FEV1: forced expiratory volume within 1 s; NSCD: non-sequential compression device; SCD: sequential compression device; VTE: venous thromboembolism.

### Preoperative diagnostic imaging

A total of 85% of the patients were node negative (N0) by computed tomography (CT) (*P *=* *0.028) and 82.5% by positron emission tomography (*P *=* *1.0) (Table 2).

**Table 2:** Diagnostic *fluorodeoxyglucose–positron emission tomography* and computed tomography oncology imaging

Characteristic^a^	Non-SCD (n = 100)	SCD (n = 100)	*P*-Value
CT-Thorax	99	100	1.0
With contrast	2	6	0.27
N-Staging			
Nx	1	0	1.0
N0	91	79	0.028
N1	5	10	0.28
N2	3	7	0.33
N3	0	4	0.12
PET scan	90	96	0.16
SUV1°	5.95 ± 5.73	5.83 ± 5.47	0.87
N-Staging			
Nx	10	4	0.16
N0	82	83	1.0
N1	5	7	0.76
N2	3	4	1.0

a Categorical and continuous data are presented as n or mean ± SD, respectively.

FDG-PET: fluorodeoxyglucose-positron emission tomography; CT: computed tomography; N: node; SCD: sequential compression device; SU: standardized uptake value.

### Caprini model venous thromboembolism risk assessment

**Figure 1:**
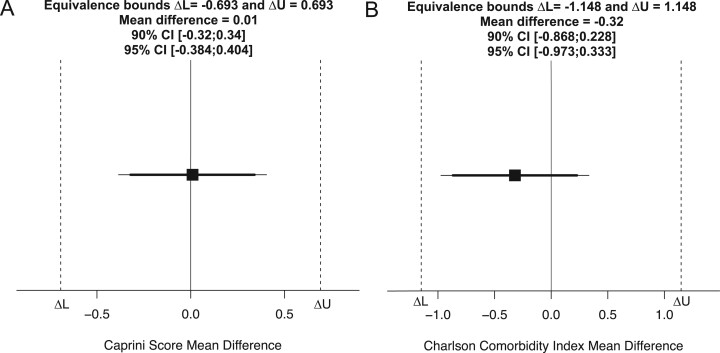
(**A**) Caprini score 2 one-sided *t*-test equivalence independent samples *t*-test analysis (*P *<* *0.001). (**B**) Charlson Comorbidity Index 2 one-sided *t*-test equivalence independent Welches *t*-test analysis (*P *<* *0.001). The raw mean difference (black square), 90% CI (thick horizontal line), 95% CI (thin horizontal line) and Cohen’s *d* [-0.49; 0.49] equivalence bounds (dark vertical dotted lines) are represented on a raw score scale. Both equivalence tests conclude that the observed mean difference is statistically not different from zero and statistically equivalent to zero, rendering the venous thromboembolism risk and burden of disease between cohorts equivalent.

The Caprini model stratified 86.5% of the patients into the high-higher VTE risk categories where the average CS between the NSCD and SCD cohorts indicate a similar high risk of VTE confirmed by a *t*-test [*t*(198) = 0.050, *P *=* *0.96] (Table 3). The TOST equivalence analysis (Fig. 1A) reports a statistically significant difference between the upper [*t*(198) = -3.414, *P *<* *0.001] and lower [*t*(198) = 3.514, *P *<* *0.001] equivalence bounds where the corresponding 90% and 95% CIs span these margins.

**Table 3:** Caprini venous thromboembolism risk model and Charlson Comorbidity Index model

Variables^a^	Non-SCD (n = 100)	SCD (n = 100)	*P*-Value
Caprini score	6.93 ± 1.31	6.92 ± 1.51	0.96
Caprini category			
Lowest (0)	0	0	…
Low (1–2)	0	0	…
Moderate (3–4)	1	3	0.62
High (5–6)	34	39	0.55
Higher (7–8)	55	45	0.20
Highest (>8)	10	13	0.65
CCI score	3.82 ± 2.04	4.14 ± 2.61	0.33

a Categorical and continuous data are presented as n or mean ± SD, respectively.

CCI: Charlson Comorbidity Index; SCD: sequential compression device.

### Charlson comorbidity index assessment

Table 3 describes an equal burden of comorbidities between cohorts prior to surgery and confirmed by a Welches *t*-test (due to unequal variances) [*t*(187.09) = 2.499, *P *=* *0.35]. The TOST equivalence analysis (Fig. 1B) reports a statistically significant difference between the upper [*t*(187.09) = -4.431, *P *<* *0.001] and lower [*t*(187.09) = 2.498, *P *<* *0.001] equivalence bounds where the corresponding 90% and 95% CIs span these margins.

### Perioperative characteristics

Outcomes pertaining to preoperative, intraoperative and postoperative phases were overall statistically not different between the cohorts (Table 4). Notably, duration of surgery (*P *=* *0.81) and LOS (*P *=* *0.60) did not vary by SCD application. The most common type of resections included lobectomy (50%) and wedge (41.5%) resections. VATS was implemented in 98–99% of cases. There was no significant difference in adverse events between the 2 groups. Within the postoperative period one patient in the SCD cohort died of pneumonia. Postoperative adverse events comprised 2 cases of atrial fibrillation within the SCD group. Emphysema, pneumothorax, pleural effusions and air leaks were recorded as pulmonary adverse events. One patient within the SCD cohort was admitted to the intensive care unit for haemoptysis. Two patients with SCDs developed transient haematuria. Thirty-day follow-up adverse events comprised 2 major generalized seizures within the SCD group with similar pulmonary adverse events between cohorts.

**Table 4:** Perioperative characteristics

Characteristics^a^	Non-SCD (n = 100)	SCD (n = 100)	*P* Value
Prior surgery (arthroscopic, major, minor, laparoscopic)	92	87	0.35
Central venous catheter	1	0	1.0
Preoperative metastatic disease	10	11	1.0
Duration of surgery, min	130.83 ± 63.73	132.85 ± 61.05	0.81
Type of resection			
Lobectomy	45	55	0.20
Segmentectomy	10	7	0.61
Wedge	45	38	0.38
Surgical approach			
VATS	99	98	1.0
Open	1	2	1.0
Location of resection			
Right upper lobe	32	28	0.64
Right middle lobe	6	6	1.0
Right lower lobe	20	18	0.85
Left upper lobe	18	27	0.17
Left lower lobe	23	13	0.097
Intraoperative chemical thromboprophylaxis			
LMWH	1	0	1.0
Rivaroxaban	0	1	1.0
Unfractionated heparin	0	1	1.0
Intraoperative complications			
Significant bleeding	0	3	0.24
Pulmonary artery injury	1	0	1.0
Staple misfire	0	1	1.0
Intensive care unit disposition	0	2	0.49
Postoperative adverse events			
Cardiac	4	4	1.0
Gastrointestinal	0	0	…
Neurological	0	0	…
Renal	0	2	0.49
Pulmonary	10	10	1.0
Death	0	1	1.0
LOS, Days	3.31 ± 3.72	3.58 ± 3.41	0.60
LOS, Days (median [interquartile range])	3 [3–4]	2 [2–4]	
30-Day follow-up adverse events			
Cardiac	0	0	…
Gastrointestinal	0	0	…
Neurological	0	2	0.49
Renal	0	0	…
Pulmonary	2	6	0.27
Death	0	0	…

a Categorical and continuous data are presented as n or mean ± SD, respectively.

LMWH: low-molecular-weight heparin; LOS: length of stay; SCD: sequential compression device; VATS: video-assisted thoracoscopic surgery,

### Pathology tumour-node-metastasis 8th edition lung cancer staging

Primary lung cancer (85%) was reported to be comparable between cohorts (*P *=* *0.30) with the predominant histological diagnosis being adenocarcinoma (67.5%) (Table 5). Staging follows the tumour-node-metastasis *EighthEditionLung Cancer Stage Classification* [21]. The most significant differences in tumour pathology were T1b (*P *=* *0.008) and T1c (*P = *0.003). Lymph node pathology did not differ across cohorts. Metastases (pulmonary metastasectomy) were confirmed in 15.5% of all patients. In each cohort, 43–52% of patients were diagnosed with stage IA. Patients with locally advance stages (IIIA) were deemed resectable at multidisciplinary oncology rounds and were followed with appropriate treatment regimens and vigilance.

**Table 5:** Pathology tumour-node-metastasis 8th edition lung cancer staging

Characteristic^a^	Non-SCD (n = 100)	SCD (n = 100)	*P* Value
Primary lung cancer	83	87	0.30
Histological diagnosis			
Adenocarcinoma	65	70	0.54
Squamous cell carcinoma	11	9	0.81
NSCLC^b^	3	12	0.065
Large cell carcinoma	0	0	…
Other	3	0	0.24
Completeness of resection			
R0	99	98	1.0
R1	1	2	1.0
Tumour pathology			
Tis	1	0	1.0
T1mi	3	0	0.24
T1a	13	17	0.55
T1b	21	39	0.008
T1c	9	0	0.003
T2a	21	17	0.58
T2b	3	5	0.72
T3	6	5	1.0
T4	1	3	0.62
Lymph node pathology			
Nx	6	5	1.0
N0	65	71	0.44
N1	4	8	0.37
N2	5	2	0.44
Cancer staging			
0	6	0	0.029
IA	43	52	0.25
IB	22	16	0.36
IIA	0	2	0.49
IIB	6	10	0.43
IIIA	6	6	1.0
IV	0	0	…
Metastases (pulmonary metastasectomy)	17	14	0.69

a Categorical data are presented as n.

b NSCLC: non-small-cell lung cancer; no further subtyping was performed.

SCD: sequential compression device.

### Postoperative and follow-up venous thromboembolism events

There was no incidence of postoperative DVT and PE between cohorts. One patient with SCD developed massive bilateral PEs on POD 2 and was admitted to the ICU for shortness of breath; the patient was treated with dalteparin; the PE resolved within 9 days. There was no incidence between the cohorts for DVT at the 30-day follow-up. Another patient with an SCD developed a 90-day follow-up PE confirmed by the CT-PE protocol on POD 43 and attenuated by 18,000 U of subcutaneous dalteparin within 7 days. There were no VTE-related deaths.

## DISCUSSION

This study is the first to date that assessed the objective quality that sequential compression devices offer with respect to minimizing VTE risk in the thoracic cancer patient population following an ERAS protocol of care. The study showcases evidence of considerable strength that suggests omitting routine use of intraoperative SCDs, provided that patients have been integrated into a strategic pathway.

The lung cancer patient population scored consistently within the high CS range of 5 to 8, facing a 1.8 to 4.0% risk of VTE, with a strong precautionary recommendation to administer SCDs (scores 0–9) and anticoagulants (scores 5–9) [22–24]. Despite the high risk of morbidity and mortality of these complex procedures and patients, the incidence and severity of postoperative VTEs were minimal and were attributed to the ERAS pathway. Of the 200 patients undergoing pulmonary resection, 2 patients in the SCD group developed PE complications. One of these patients developed massive bilateral PEs on POD 2 within the 30-day follow-up period. This patient was treated with dalteparin; the PE resolved on POD 11. The other patient developed a PE on POD 43 and was treated with subcutaneous dalteparin; the PE resolved on POD 50. These patients had average CSs of 6 and 8, with CCIs of 3 and 4, respectively. They were both aged about 70 years; the former patient had hypertension and the latter, chronic obstructive pulmonary disease. Their preoperative VTE risk assessment aligns with that of patients within the NSCD cohort who faced no incidence of DVT or PE. These 2 patients did not suffer from any previous cancer, had standard creatinine levels with a pack-year history of 22 and 75, respectively. They underwent a VATS lobectomy of the right upper lobe or the left lower lobe, with a pathological stage of IIB and histological diagnosis of adenocarcinoma in both cases. Their corresponding LOS was 5 and 11 days, respectively. PE was assessed by CT angiography upon presentation to the emergency department. Aside from these PE events, there were no incidences of symptomatic DVT postoperatively or within the 30 and 90 days following discharge; nor were there any VTE-related deaths. It is evident that SCDs did not offer any major support in preventing VTEs in these 2 patients and that intrinsic factors related to their progressed staging may have predisposed them to this condition.

The TOST equivalence analysis demonstrated an equal burden of comorbidities (CCI) and VTE risk factors (CS) between the cohorts (Fig. 1). Given the low incidence of VTE between the cohorts, addressing embolic risk through an SCD modality is not warranted based solely on CCI and CS metrics when both cohorts have statistically equivalent risk factors and characteristics. Shargall *et al.* conducted a recent double-blind placebo-controlled randomized trial assessing extended pharmacological prophylaxis following major lung cancer resection. Their findings support guideline recommendations in extending pharmacological prophylaxis to 30 days after the operation [25]. The American Society of Hematology 2021-VTE guideline reports a conditional recommendation in extending postoperative LMWH (up to 4 weeks) compared to limited thromboprophylaxis (7–10 days post-discharge), given that the risk of VTE persists long after the operation [26]. However, this recommendation was limited by abdominal and pelvic surgical studies. Furthermore, a meta-analysis of 12 trials reported the concern of SCDs being overused and inferior to anticoagulants [27]. It is therefore warranted to suggest examining a postoperative course of extended chemical thromboprophylaxis in an ERAS patient population following discharge.

### Limitations

The findings of this study are limited by the retrospective selection bias of patients receiving intraoperative SCDs and analysis restricted to symptomatic VTEs where asymptomatic occurrence was not evaluated by a CT-PE protocol. It is the gold standard for implementing randomized controlled trials in allocating SCDs; however, to begin assessing such an intervention in high-risk thoracic oncology patients warrants a preliminary study. Due to the underpowered sample size, there may be a limited incidence of VTEs because it may present in a wider array and more diverse sample and may explain the increased frequency of 30-day adverse events in the SCD cohort. A total of 85% of patients presented with early-stage lung cancer. As a result, the findings of this study are limited to this cohort of patients who predominantly underwent VATS lobectomy. There is a difference between the sex ratio of males:females (2:3) undergoing pulmonary resection; however, we find this difference reflects current patient trends. These limitations are amenable to larger sample sizes and multicentre collaborations to encompass the diversity of characteristics in this patient population.

### Conclusion

Perioperative care guided by ERAS protocols mediates the postoperative incidence of DVT and PE despite the moderate-high CS and CCI values in high-risk thoracic surgical patients. Although further evidence is needed, our data suggest that, based solely on these metrics, routine intraoperative SCD use in patients with VATS-resected early-stage lung cancer may not always be required, encouraging reticence in intraoperative SCD use and re-evaluating this recommendation in the thoracic ERAS guideline. Studies including ours warrant examining extended pharmacological thromboprophylaxis to 30 days post-discharge. Establishing evidence concerning the use of SCDs reduces unnecessary costs, optimizes ERAS, contributes to evidence-based medicine and improves the quality of surgical care.

## Notes

**Meeting presentation:** Presented at the Canadian Surgery Forum: Canadian Association of Thoracic Surgeons (CSF-CATS) Annual Meeting, Virtual, Canada, September 21-24, 2021.

Poster Presentation at the American Association of Thoracic Surgeons, 102nd Annual Meeting, Boston, MA, USA, May 14-17, 2022.

## Abbreviation and acronyms

CCI: Charlson Comorbidity Index
CI: Confidence interval
CT: Computed tomography
DVT: Deep vein thrombosis
ERAS: Enhanced recovery after surgery
LMWH: Low-molecular-weight heparin
LOS: Length of stay
NSCD: Non-sequential compression device
PE: Pulmonary embolism
POD: Postoperative day
SCD: Sequential compression device
TOST: Two one-sided *t*-test
VTE: Venous thromboembolism
